# Attention, attention! Posttraumatic stress disorder is associated with altered attention-related brain function

**DOI:** 10.3389/fnbeh.2023.1244685

**Published:** 2023-08-21

**Authors:** Samantha L. Ely, Clara G. Zundel, Leah C. Gowatch, Julia M. Evanski, Amanpreet Bhogal, Carmen Carpenter, MacKenna Shampine, Hilary Marusak

**Affiliations:** ^1^Department of Psychiatry and Behavioral Neurosciences, Wayne State University School of Medicine, Detroit, MI, United States; ^2^Translational Neuroscience PhD Program, Wayne State University School of Medicine, Detroit, MI, United States; ^3^Neuroscience Graduate Program, University of Michigan, Ann Arbor, MI, United States; ^4^Department of Pharmacology, Wayne State University School of Medicine, Detroit, MI, United States; ^5^Merrill Palmer Skillman Institute, Wayne State University, Detroit, MI, United States

**Keywords:** posttraumatic stress, attention network, veterans, trauma, resting-state, continuous performance task, functional magnetic resonance imaging, neuroimaging

## Abstract

Posttraumatic stress disorder (PTSD) is a debilitating condition characterized by altered arousal, mood, and cognition. Studies report attentional alterations such as threat bias in individuals with PTSD, though this work has largely been conducted within emotionally-charged contexts (e.g., threatening stimuli). Emerging behavioral evidence suggests that PTSD-related attention deficits exist even in the absence of threatening cues or anxiety triggers. However, the role and functioning of attention brain circuits as they relate to PTSD remains underexplored. In this mini review, we highlight recent work using non-emotional stimuli to investigate the neurobiology of attention and disruptions to attention-related brain function among individuals with PTSD. We then discuss gaps in the current literature, including questions pertaining to the neural circuitry of attentional alterations in PTSD, as well as the contributions that trauma exposure, PTSD symptoms, comorbidities, and pre-existing vulnerabilities may have in this relationship. Finally, we suggest future directions for this emerging area of research, which may further inform knowledge surrounding the neurobiological underpinnings of PTSD and potential treatments.

## 1. Introduction

Life stressors fog the mind, diverting attention away from daily functions. Typically, this foggy feeling dissipates quickly. However, this feeling can persist in the presence of severe stressors (e.g., traumatic events), as is the case for individuals with posttraumatic stress disorder (PTSD). Criterion A traumatic events encompass “actual or threatened death, serious injury, or sexual violence,” (American Psychiatric Association [APA], 2013:271), including physical/sexual assault, disasters, and war exposure. Lifetime trauma incidence varies worldwide, ranging 28–85% (Benjet et al., 2016), whereas prevalence of PTSD ranges 6.8–9.2% (Kessler et al., 2005; Dückers et al., 2016).

Posttraumatic stress disorder symptoms include intrusive thoughts, avoidance behaviors, hyperarousal, and negative alterations in mood/cognition (American Psychiatric Association [APA], 2013). This debilitating disorder frequently co-occurs with other disorders (Brady et al., 2000) and causes substantial burden, resulting in poor psychosocial (Kuhn et al., 2003) and physical function (Ahmadian et al., 2019). PTSD also impacts society, with an estimated $232.2 billion yearly excess economic burden in the U.S. (Davis et al., 2022). Ostensibly, PTSD has far-reaching effects persisting long after the traumatic event.

First-line PTSD treatments include pharmacologic (e.g., paroxetine, sertraline), and psychological approaches (e.g., prolonged exposure). While current treatments reduce symptoms for many (Hoskins et al., 2015; Watkins et al., 2018), improvements are needed due to small treatment effect sizes (Cipriani et al., 2017), frequent diagnosis retention (Steenkamp et al., 2015), and poor acceptability (Lewis et al., 2020). These limitations may reflect substantial heterogeneity in PTSD (Galatzer-Levy and Bryant, 2013), and/or that current treatments may not target underlying aspects or subtypes of PTSD (Susanty et al., 2022).

Recent research highlights the potential role of altered *attention* in PTSD (Block and Liberzon, 2016; Punski-Hoogervorst et al., 2023). Attention is “the range of processes that regulate access to capacity-limited systems, such as awareness, higher perceptual processes, and motor action” (National Institute of Mental Health, 2021). Attentional abnormalities could contribute to hyperarousal and intrusive PTSD symptoms, manifesting as emotional reactivity or inattention (Block and Liberzon, 2016). Investigating cross-cutting characteristics of psychiatric disorders, like attention, can improve knowledge of the development, expression, and treatment of PTSD (Scott et al., 2015). Nevertheless, compared to related constructs like memory (Johnsen and Asbjørnsen, 2008), attention-related brain circuits as they relate to PTSD remain underexplored. To date, PTSD research predominantly examines attentional processes under threat cues (Pergamin-Hight et al., 2015) and other emotionally-charged contexts (e.g., neurobehavioral attention biases toward fearful faces Fani et al., 2012). However, a recent systematic review of neuropsychological studies suggests that PTSD-related behavioral attention deficits remain even in neutral settings without emotional stimuli (Punski-Hoogervorst et al., 2023), though the neuroanatomical underpinnings of this relationship are under-researched.

Here, we review current literature on attention-related brain function in individuals with PTSD. First, we briefly overview attention-related brain networks and two well-validated tasks used to study attentional processes. We then summarize recent functional magnetic resonance imaging (fMRI) studies highlighting altered attention-related brain function associated with PTSD symptoms in the absence of emotional cues. Finally, we discuss gaps in the literature and offer future research recommendations.

## 2. Attention-related brain networks

Across disciplines, attention has been modeled under various frameworks (see Lindsay, 2020 for a review). Electrophysiological studies in non-human primates provide evidence for signal detection theory, suggesting that distinct neuronal mechanisms contribute to different behavioral aspects of attention (e.g., sensitivity and response to stimuli; Luo and Maunsell, 2019). In humans, neurobehavioral studies support a model of three anatomically and functionally distinct attention networks (alerting, orienting, and executive) involving different neuromodulatory systems and brain regions (Petersen and Posner, 2012). Alerting attention refers to the maintenance of an arousal state to temporally process stimuli, while orienting attention describes the ability to spatially attune toward stimuli. Executive attention enables voluntary attentional control, as well as conflicting stimuli detection and resolution.

**FIGURE 1:**
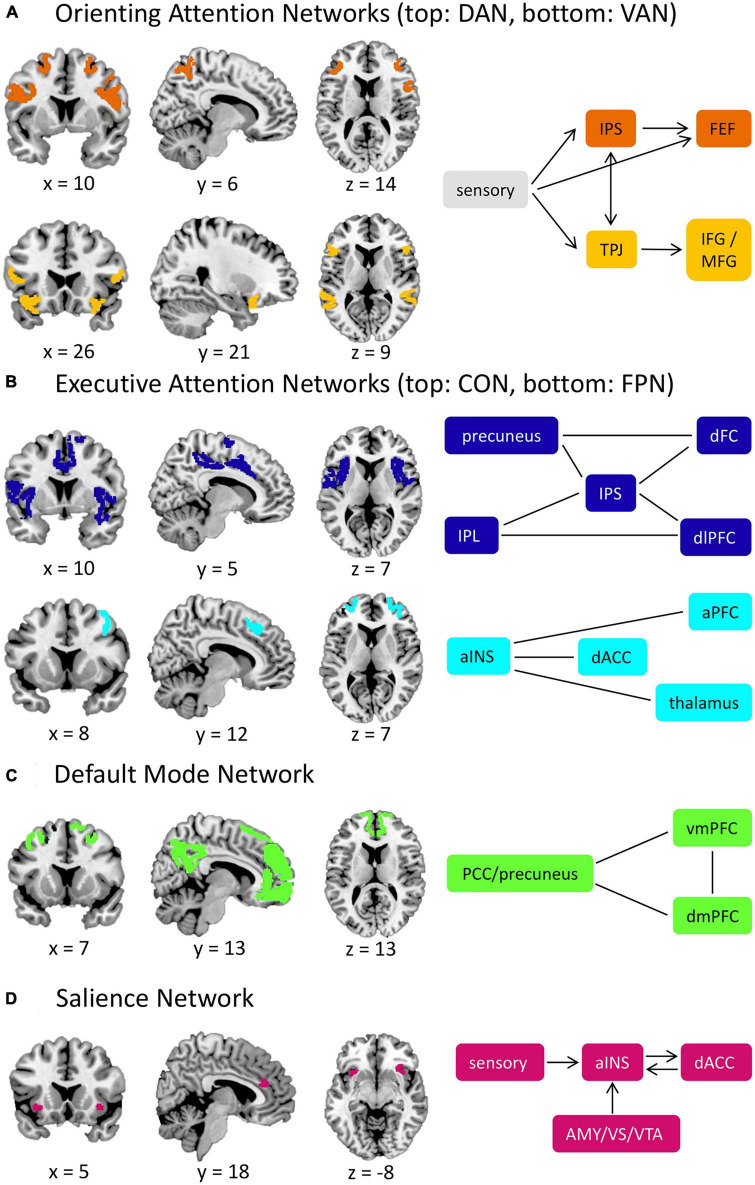
Attention-related brain networks. **(A)** Orienting attention networks include the DAN and VAN. The DAN is involved in top-down control of attention, while the VAN is responsible for bottom-up attention. When external salient stimuli are detected, the VAN will interrupt the processes of the DAN through the TPJ. **(B)** Executive attention networks include the CON and FPN. The CON provides a stable maintenance of attention performance, and the FPN is responsible for task initiation and switching. **(C)** The DMN is involved in self-reflective mental activity and is typically less active during externally oriented tasks, such as tasks that require attentional control. **(D)** The SN is involved in detecting salient stimuli (including interoceptive stimuli) and facilitates the switch between the FPN and the DMN. aINS, anterior insula; AMY, amygdala; aPFC, anterior prefrontal cortex; CON, cingulo-opercular network; dACC, dorsal anterior cingulate cortex; DAN, dorsal attention network; dFC, dorsal frontal cortex; dlPFC, dorsolateral prefrontal cortex; DMN, default mode network; dmPFC, dorsomedial prefrontal cortex; FEF, frontal eye fields; FPN, frontoparietal network; IFG, inferior frontal gyrus; IPL, inferior parietal lobule; IPS, intraparietal sulcus; MFG, middle frontal gyrus; PCC, posterior cingulate cortex; SN, salience network; TPJ temporoparietal junction; VAN, ventral attention network; vmPFC, ventromedial prefrontal cortex; VS, ventral striatum; VTA, ventral tegmental area. Straight lines represent connectivity between structures in cases where there is not a definitive directional relationship. Network nodes derived from the Gordon network parcellation scheme (Gordon et al., 2016).

Norepinephrine facilitates alerting attention with projections from the locus coeruleus (Aston-Jones and Cohen, 2005) to various brain regions in the parietal and frontal cortices (Petersen and Posner, 2012). Acetylcholine modulates orienting attention (Petersen and Posner, 2012) through the dorsal (DAN) and ventral attention networks (VAN; Corbetta and Shulman, 2002; Figure 1A). The DAN exerts top-down attentional control and includes the intraparietal sulcus and frontal eye fields. In contrast, the VAN facilitates bottom-up control to direct attention toward salient stimuli and is anchored by the temporoparietal junction and inferior/middle frontal gyri. Together, these networks interact dynamically in service of flexible attentional control (Vossel et al., 2014).

Dopamine and serotonin govern executive attention under two distinct networks: the cingulo-opercular (CON) and frontoparietal networks (FPN; Dosenbach et al., 2008; Figure 1B). The CON provides stable surveillance of attentional performance and involves the dorsal anterior cingulate cortex (dACC), anterior insula, and thalamus. The FPN, anchored by the dorsolateral prefrontal cortex and inferior parietal lobule, initiates and adjusts attention and may also control spatial orienting and emotional regulation processes (Dosenbach et al., 2008; Scolari et al., 2015). Opposing these executive attention networks is the default-mode network (DMN; Figure 1C), which deactivates during tasks and activates during rest periods. The DMN facilitates internally-directed mental activity and includes the ventromedial/dorsomedial prefrontal cortices and posterior cingulate cortex/precuneus (Raichle, 2015).

The salience network (SN; Figure 1D) shares a similar role to the VAN in detecting salient stimuli (e.g., threats, rewards). The anterior insula and dACC anchor the SN, but can also incorporate subcortical structures (e.g., the amygdala). Notably, the SN, VAN, and CON are sometimes used interchangeably in the literature, likely due to their closely related roles and anatomical overlap (Gratton et al., 2018). However, evidence suggests that these networks are functionally distinct (Seeley, 2019; Uddin et al., 2019). Specifically, the SN recruits task-control networks and facilitates the switch between the FPN and DMN, whereas the CON provides stable task performance monitoring (Seeley et al., 2007; Menon, 2015; Seeley, 2019). The SN also broadly detects salient information, including interoceptive stimuli, whereas the VAN detects exogenous stimuli (Uddin et al., 2019). For further discussion, see Gratton et al., 2018; Seeley, 2019; Uddin et al., 2019.

## 3. Attention-related brain and behavioral tasks

**FIGURE 2:**
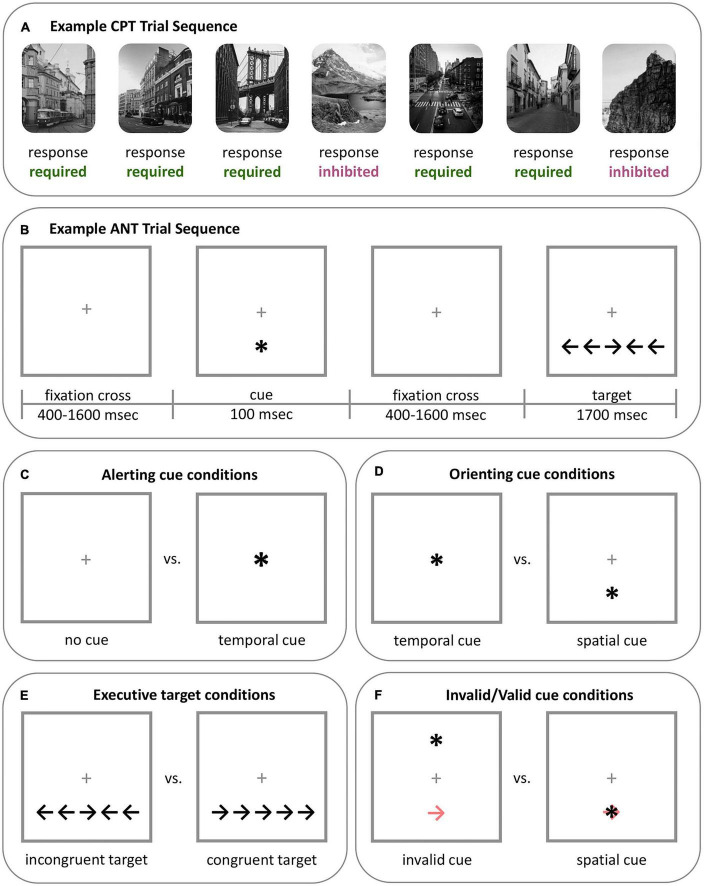
Example of two well validated tasks that assess attention. **(A)** Example CPT sequence. The CPT includes two types of stimuli: stimuli that require a response (button press), which are most frequent (e.g., 80% of trials), and stimuli that require inhibition, which are less frequent (e.g., 20% of trials). In this example sequence, city scenes require a response while mountain scenes require inhibition. The type of stimuli and aspects of the task can vary. For example, a gradual-onset CPT (gradCPT), such as the one used in Evans et al. (2022), can have stimuli fade from one to the other, rather than appearing separately or with a fixation cross between stimuli. The CPT measures sustained attention. Participants’ ability to sustain attention can be measured by factors such as accuracy and variability in the RT to stimuli requiring a response across the task. Response strategy can be assessed through the mean RT of the task and criterion (omission and commission errors). This figure was adapted from Evans et al. (2022). **(B)** Example ANT sequence. In the ANT, participants respond by indicating the direction of the central target arrow (i.e., to the left or to the right) that will appear at the top or bottom of the screen. Trials may or may not include a preceding warning cue (“central cue”), and if present, this warning cue may indicate the location of the following target (“spatial cue”). Participants’ reaction to the target on various trials is used to measure attention network performance. **(C)** Alerting attention is measured by contrasting participants’ RT to the target during trials with no warning cue and trials with a central warning cue (i.e., RT_no_ _cue_ - RT_central_). **(D)** Orienting attention is measured by contrasting participants’ RT to the target during trials with a central warning cue and trials with a spatial cue (i.e., RT_central_ - RT_spatial_). **(E)** Executive attention is measured by contrasting participants’ RT to the target during trials where the target appears with incongruent flankers and trials with congruent flankers (i.e., RT_incongruent_ - RT_congruent_). **(F)** The validity effect appears in adapted versions of the ANT and is used to measure the ability to disengage from spatial cues, or if the spatial cues are being utilized by the participant. The validity effect is determined by contrasting participants’ RT to trials with invalidly indicated spatial cues (which appear in the opposite location of the target; light red arrow) with trials that have a validly indicated spatial cue (i.e., RT_invalid_ - RT_valid_). *ANT, Attention network test; CPT, continuous performance task; RT, reaction time.

Several fMRI tasks measure attention-related brain and behavioral responses across different domains (see Tables 3, 4 in Block and Liberzon, 2016). Widely used, continuous performance tasks (CPT) capture sustained attention through repeated stimuli presentation over long time periods (Figure 2A). During a CPT, participants respond to target stimuli while inhibiting responses to others. In line with signal detection theory, the CPT can provide separable measures of participants’ ability to detect target stimuli (i.e., sensitivity) and appropriate initiation or inhibition toward stimuli (i.e., response; Evans et al., 2022).

The Attention Network Test (ANT; Fan et al., 2002) concurrently evaluates alerting, orienting, and executive attention networks (Figures 2B–E). In the ANT, participants identify the direction of a central target arrow, which is flanked by cues facing the same or opposite direction and measures executive attention. Some trials are preceded by a warning cue to signal the incoming target and may indicate its future location, which measures alerting and orienting attention, respectively.

## 4. Studies of altered attention-related brain function associated with PTSD

Here, we review four fMRI studies highlighting the role of non-emotionally driven attention in PTSD.

### 4.1. Block et al. (2017)

In this study, 49 male combat-exposed veterans with PTSD and 26 healthy control (HC) male civilians completed the ANT. Block et al. (2017) found no alerting or executive attention differences between groups; however, they reported individuals with PTSD had impaired orienting attention relative to HC. Further, orienting attention performance negatively correlated with intrusive symptoms of PTSD. The authors suggest that PTSD’s effect on orienting attention may indicate an impairment in disengaging from cues, which may contribute to PTSD-related threat biases (Aupperle et al., 2012), though this disengagement concern encompasses non-emotional stimuli as well. In addition, the authors explored the association between DAN resting-state functional connectivity (rs-FC) and orienting attention performance. Poorer orienting attention was associated with greater cross-network rs-FC between the middle frontal gyrus (considered a DAN node in this study) and the right amygdala (SN). However, only the HC group significantly displayed this relationship, suggesting the absence of an expected brain-behavior correlation in the PTSD group. Independent of attentional performance, the PTSD group exhibited higher rs-FC between the DAN (middle frontal gyrus) and the SN (right amygdala). Together, the authors suggest that these findings indicate impaired orienting attention and a disruption in attention-related brain networks (i.e., DAN, SN) in PTSD compared with HC.

### 4.2. Block et al. (2020)

The previous study showed general alterations in orienting attention and underlying brain circuits among individuals with PTSD. To confirm whether this effect stemmed from difficulties in disengaging from spatial cues (or reflected different utilization of cues), Block et al. (2020) had 31 individuals with PTSD and 21 HC (community sample, 93% female) complete an adapted ANT. The adapted ANT includes an invalid cue appearing in the opposite location of the target, and participants’ responses provide information on spatial cue disengagement/utilization (the “validity effect”; Figure 2F). Compared with HC, those with PTSD showed a smaller validity effect, indicating lower spatial cue utilization to inform subsequent behavior. Task-based fMRI data collected during the ANT supported a brain-behavior correlation between the validity effect and SN activity (right insula) during orienting trials. Interestingly, this relationship significantly differed between groups: lower spatial cue utilization was associated with higher SN activity in the PTSD group and lower activity in the HC group. Notably, this study included a third group of trauma-exposed controls without PTSD. However, this group did not differ from the PTSD or HC groups in validity effect or its association with SN activity, suggesting that spatial attention deficits may be associated with trauma exposure itself (Block et al., 2020).

### 4.3. Esterman et al. (2020)

In a sample of predominantly male (90%) combat-exposed veterans with (*n* = 140) and without (*n* = 89) PTSD, Esterman et al. (2020) examined the relationship between attention-related behavior and rs-FC of seven core networks (Yeo et al., 2011). Composite scores of attentional performance were generated using neuropsychological tasks. Results showed more individuals with PTSD had clinical-level attentional impairments compared to those without PTSD. Those with PTSD and attentional impairments demonstrated lower within-VAN rs-FC relative to those without attentional impairments and those without PTSD. Further, among this subgroup, lower composite scores (greater impairments) were correlated with lower within-VAN rs-FC. Interestingly, these effects were specific to attention and no other cognitive domains (e.g., verbal memory). Together, these findings indicate a PTSD neurocognitive subtype characterized by attentional impairments and lower within-VAN rs-FC.

### 4.4. Evans et al. (2022)

Evans et al. (2022) conducted several studies examining the neural circuitry underlying PTSD-related attentional impairments. First, two independent samples of post-9/11 veterans (n_total_ = 327, ∼90% male) completed the gradual-onset CPT (gradCPT), which assesses sustained attention ability and response strategy (e.g., impulsive responding). They found greater PTSD symptoms were associated with lesser sustained attention ability, but not impulsive response strategies.

To disentangle the neural correlates of this finding, fMRI was used in a third independent sample of 117 post-9/11 veterans (∼92% male). The authors hypothesized that PTSD-related sustained attention differences may reflect a dysregulation of neural systems that: (1) support effortful and/or automatic control during active attentional demands, or (2) are global and not necessarily specific to sustained attention demands. The latter hypothesis was based on recent neurobiological models suggesting that PTSD symptoms are associated with global patterns of dysfunction within large-scale neurocognitive networks—particularly the SN, DMN, and FPN (the “triple network hypothesis”)–even during resting-state conditions (Akiki et al., 2017; Ross and Cisler, 2020). To examine these hypotheses, the authors measured PTSD symptoms’ impact on brain-behavior synchronization during the gradCPT and during resting-state. Attentional fluctuations were measured by reaction time (RT) variability across the gradCPT, wherein higher RT variability periods reflect suboptimal attention and lower RT variability periods reflect more optimal attention. Importantly, sustained attention fluctuations vary with neural network activity, specifically in the DAN (i.e., brain-behavior synchronization; Esterman et al., 2014). In other words, attention fluctuates across time, and these behavioral fluctuations correspond with DAN activity. Results showed greater PTSD symptoms were associated with weaker positive DAN synchronization which was, in turn, associated with impaired sustained attention. Interestingly, PTSD symptoms were not associated with task-based activity or rs-FC within or between the SN, DMN, and FPN. These findings suggest that PTSD symptoms selectively impair sustained attention ability and DAN engagement, rather than correspond with a more global pattern of dysfunction.

## 5. Discussion and summary

Recent research links PTSD to altered attention-related behavior, even when emotional stimuli are absent (Punski-Hoogervorst et al., 2023); however, the current neuroanatomical understanding of this relationship is limited. Here, we examined four recent studies that highlight attention-related brain function in individuals with PTSD symptoms in the presence of non-emotional cues. Findings indicate that PTSD symptoms are associated with poorer orienting attention (Block et al., 2017), possibly due to lesser spatial cue utilization (Block et al., 2020), suboptimal sustained attention performance (Evans et al., 2022), and/or overall attentional impairment (Esterman et al., 2020). FMRI analyses showed that PTSD symptoms are associated with altered brain-behavior correlations within or between three core attentional-control networks: the DAN, VAN, and SN. Together, these studies support a general impairment in fundamental attentional processes and altered network function in individuals with PTSD symptoms.

The behavioral data evidence PTSD-related sustained and orienting attention deficits. Evans et al. (2022) suggest that PTSD-related sustained attention deficits may contribute to functional impairments (e.g., maintaining employment). Block et al. (2020) propose that PTSD-related effects on orienting attention contribute to contextual processing differences, potentially explaining the abnormal fear responses in neutral, non-threatening environments reported in PTSD. Altered visual processing may disrupt attention networks and contribute to these difficulties (or vice versa; Mueller-Pfeiffer et al., 2013). Interestingly, recent evidence links structural ventral visual stream covariance to flashback and nightmare symptoms after a traumatic event, and the integrity of these areas predicts future PTSD symptom severity (Harnett et al., 2022). Future studies should investigate the role of visual processing in attention-related brain function and behavior in individuals with PTSD.

All reviewed studies reported PTSD-related alterations within and between crucial top-down and bottom-up attentional control networks (i.e., the DAN, VAN, and SN). Evans et al. (2022) findings of PTSD-related reductions in DAN synchronization are intriguing, considering the DAN’s top-down control over visuospatial attention. Individuals with PTSD in Block et al.’s studies (2017, 2020) exhibited (1) an absent but expected brain-behavior correlation between orienting attention and DAN-SN rs-FC and (2) a relationship between the validity effect and SN activity. Given the DAN, VAN, and SN orchestrate attentional resources (Corbetta et al., 2008), these findings suggest that salient stimuli detected by the VAN/SN may fail to appropriately redirect attentional control by interrupting the DAN’s processes in individuals with PTSD. These networks also control the DMN, with the DAN/SN suppressing activity (Zhou et al., 2018) and the SN facilitating the FPN-DMN switch (Uddin, 2014). Therefore, these network alterations may indicate an inability to redirect attention away from internal stimuli and toward exogenous demands. Future studies should further investigate this phenomenon.

Interestingly, SN dysfunction may be a shared feature across disorders, as meta-analyses comparing patients with various Axis I disorders to HC reveal consistent patterns of gray matter loss and abnormal activation in the insula and other SN nodes (Goodkind et al., 2015; McTeague et al., 2017). Considering PTSD frequently co-occurs with other disorders (Brady et al., 2000), the reported effects of PTSD on attention-related brain-behavior function could represent cross-cutting psychopathology. Additionally, PTSD-related effects on attentional processes may be due to significant symptom heterogeneity (Galatzer-Levy and Bryant, 2013), suggesting subtypes may better characterize PTSD and treatment response (France and Jovanovic, 2023). Esterman et al. (2020) identified a PTSD subtype hallmarked by attentional impairments and lower within-VAN rsFC, potentially contributing to other cognitive disruptions (e.g., memory) by increasing distractions or intrusions (Vasterling et al., 1998). Furthermore, trauma-exposed controls did not differ significantly from PTSD or HC groups (c.f. Block et al., 2020), suggesting that altered attentional processes represent a pre-existing vulnerability, and/or that trauma exposure itself may induce such alterations. For example, interpersonal early-life trauma, rather than PTSD, was found to contribute to neurobehavioral attention abnormalities in a sample of veterans (Fortenbaugh et al., 2017). Together, these ideas necessitate studies to disentangle the role of trauma exposure, PTSD expression, comorbidities, and/or pre-existing vulnerabilities in attentional processes.

Attention difficulties are common in PTSD and extend beyond threat bias to affect general cognition, yet current treatments often fail to target these symptoms (Susanty et al., 2022). Further, attentional impairments may maintain symptoms and interfere with treatment response (Esterman et al., 2020). Punski-Hoogervorst et al. (2023) propose that attention-targeting treatments (e.g., methylphenidate, attention training) may fill gaps in current approaches by addressing cognitive dysfunction in PTSD. Research utilizing non-emotional stimuli would elucidate the role of cognitive attention in PTSD symptom maintenance and treatment response, and potentially enhance interventions. Used alongside emotional stimuli, this research could disentangle the cognitive and emotional mechanisms driving the complex neurobiology of PTSD.

Emerging evidence supports the link between PTSD symptoms and general attentional abnormalities; yet, several questions remain unanswered. Three out of four studies included predominantly white, male veterans, which limits the generalizability of findings. Future investigations should include individuals who are female, from diverse racial/ethnic backgrounds, and exposed to civilian trauma. The lack of research conducted during substantial attention network development (e.g., childhood; Saito et al., 2022) is significant. Notably, most individuals (61–68%) experience trauma during developmental periods (Copeland et al., 2007; McLaughlin et al., 2013), and many psychiatric disorders–including PTSD–originate during this time (De Lijster et al., 2017). Furthermore, early life trauma, particularly interpersonal trauma, impacts attentional processes with observable effects well into adulthood (Fortenbaugh et al., 2017), though trauma’s impact *during* development is under-researched. Consequently, it is imperative to investigate the impact of trauma exposure and/or PTSD on attention network development.

To date, few neuroimaging studies employing non-emotional cues to investigate attentional processes have been conducted. This review provides preliminary evidence that PTSD symptoms impact brain-behavior correlations even in the absence of emotional cues, suggesting a broad impairment in attention-related brain networks. It is crucial for future research to both examine brain-based subtypes of attentional alterations associated with PTSD and untangle the effects of PTSD, trauma exposure, comorbidities, and other significant factors such as childhood trauma. Nevertheless, this review contributes to the growing neuropsychological evidence highlighting the prevalence of attention deficits among individuals with PTSD and emphasizes the need for further research in this area to inform treatment strategies.

## Author contributions

SE and HM were involved in the initial conceptualization of the topic and produced the first drafts of the manuscript. CZ, LG, JE, AB, CC, and MS all provided valuable insights in the formation of the topic. All authors critically reviewed and revised the manuscript and figures, and approve of the manuscript’s submission.

## References

[B1] Ahmadian A. J. Neylan T. C. Metzler T. Cohen B. E. (2019). Longitudinal association of PTSD symptoms and self-reported physical functioning among veterans. *J. Affect. Disord.* 250 1–8. 10.1016/J.JAD.2019.02.048 30818050

[B2] Akiki T. J. Averill C. L. Abdallah C. G. (2017). A network-based neurobiological model of PTSD: Evidence from structural and functional neuroimaging studies. *Curr. Psychiatry Rep.* 19:81. 10.1007/S11920-017-0840-4 28924828 PMC5960989

[B3] American Psychiatric Association [APA] (2013). *Diagnostic and statistical manual of mental disorders: DSM-5*, 5th Edn. Washington, DC: American Psychiatric Association.

[B4] Aston-Jones G. Cohen J. D. (2005). An integrative theory of locus coeruleus-norepinephrine function: Adaptive gain and optimal performance. *Annu. Rev. Neurosci.* 28 403–450. 10.1146/ANNUREV.NEURO.28.061604.135709 16022602

[B5] Aupperle R. L. Melrose A. J. Stein M. B. Paulus M. P. (2012). Executive function and PTSD: Disengaging from trauma. *Neuropharmacology* 62:686. 10.1016/J.NEUROPHARM.2011.02.008 21349277 PMC4719148

[B6] Benjet C. Bromet E. Karam E. G. Kessler R. C. McLaughlin K. A. Ruscio A. M. (2016). The epidemiology of traumatic event exposure worldwide: Results from the World Mental Health Survey Consortium. *Psychol. Med.* 46:327. 10.1017/S0033291715001981 26511595 PMC4869975

[B7] Block S. R. King A. P. Sripada R. K. Weissman D. H. Welsh R. Liberzon I. (2017). Behavioral and neural correlates of disrupted orienting attention in posttraumatic stress disorder. *Cogn. Affect. Behav. Neurosci.* 17 422–436. 10.3758/s13415-016-0488-2 27966102

[B8] Block S. R. Liberzon I. (2016). Attentional processes in posttraumatic stress disorder and the associated changes in neural functioning. *Exp. Neurol*. 284, 153–167. 10.1016/J.EXPNEUROL.2016.05.009 27178007

[B9] Block S. R. Weissman D. H. Sripada C. Angstadt M. Duval E. R. King A. P. (2020). Neural mechanisms of spatial attention deficits in trauma. *Biol. Psychiatry Cogn. Neurosci. Neuroimaging* 5 991–1001. 10.1016/j.bpsc.2019.05.014 31377230

[B10] Brady K. Killeen T. Brewerton T. Lucerini S. (2000). Comorbidity of psychiatric disorders and posttraumatic stress disorder. *J. Clin. Psychiatry* 61 22–32. 10795606

[B11] Cipriani A. Williams T. Nikolakopoulou A. Salanti G. Chaimani A. Ipser J. (2017). Psychological medicine comparative efficacy and acceptability of pharmacological treatments for post-traumatic stress disorder in adults: A network meta-analysis. *Psychol. Med.* 48 1975–1984. 10.1017/S003329171700349X 29254516

[B12] Copeland W. E. Keeler G. Angold A. Costello E. J. (2007). Traumatic events and posttraumatic stress in childhood. *Arch. Gen. Psychiatry* 64 577–584. 10.1001/ARCHPSYC.64.5.577 17485609

[B13] Corbetta M. Shulman G. L. (2002). Control of goal-directed and stimulus-driven attention in the brain. *Nat. Rev. Neurosci.* 3 201–215. 10.1038/nrn755 11994752

[B14] Corbetta M. Patel G. Shulman G. L. (2008). The reorienting system of the human brain: From environment to theory of mind. *Neuron* 58:306. 10.1016/J.NEURON.2008.04.017 18466742 PMC2441869

[B15] Davis L. L. Schein J. Cloutier M. Gagnon-Sanschagrin P. Maitland J. Urganus A. (2022). The economic burden of posttraumatic stress disorder in the United States from a societal perspective. *J. Clin. Psychiatry* 83:21m14116. 10.4088/JCP.21M14116 35485933

[B16] De Lijster J. M. Dierckx B. Utens E. M. W. J. Verhulst F. C. Zieldorff C. Dieleman G. C. (2017). The age of onset of anxiety disorders. *Can. J. Psychiatry* 62 237–246. 10.1177/0706743716640757 27310233 PMC5407545

[B17] Dosenbach N. U. F. Fair D. A. Cohen A. L. Schlaggar B. L. Petersen S. E. (2008). A dual-networks architecture of top-down control. *Trends Cogn. Sci.* 12:99. 10.1016/J.TICS.2008.01.001 18262825 PMC3632449

[B18] Dückers M. L. A. Alisic E. Brewin C. R. (2016). A vulnerability paradox in the cross-national prevalence of post-traumatic stress disorder. *Br. J. Psychiatry* 209 300–305. 10.1192/bjp.bp.115.176628 27445357

[B19] Esterman M. Rosenberg M. D. Noonan S. K. (2014). Intrinsic fluctuations in sustained attention and distractor processing. *J. Neurosci.* 34 1724–1730. 10.1523/JNEUROSCI.2658-13.2014 24478354 PMC6827583

[B20] Esterman M. Stumps A. Jagger-Rickels A. Rothlein D. Degutis J. Fortenbaugh F. (2020). Evaluating the evidence for a neuroimaging subtype of posttraumatic stress disorder. *Sci. Transl. Med.* 12:9343. 10.1126/scitranslmed.aaz9343 33148627

[B21] Evans T. C. Alonso M. R. Jagger-Rickels A. Rothlein D. Zuberer A. Bernstein J. (2022). PTSD symptomatology is selectively associated with impaired sustained attention ability and dorsal attention network synchronization. *Neuroimage Clin.* 36:103146. 10.1016/J.NICL.2022.103146 36055063 PMC9437905

[B22] Fan J. McCandliss B. D. Sommer T. Raz A. Posner M. I. (2002). Testing the efficiency and independence of attentional networks. *J. Cogn. Neurosci.* 14 340–347. 10.1162/089892902317361886 11970796

[B23] Fani N. Jovanovic T. Ely T. D. Bradley B. Gutman D. Tone E. B. (2012). Neural correlates of attention bias to threat in post-traumatic stress disorder. *Biol. Psychol.* 90:134. 10.1016/J.BIOPSYCHO.2012.03.001 22414937 PMC3340884

[B24] Fortenbaugh F. C. Corbo V. Poole V. McGlinchey R. Milberg W. Salat D. (2017). Interpersonal early–life trauma alters amygdala connectivity and sustained attention performance. *Brain Behav.* 7:e00684. 10.1002/brb3.684 28523226 PMC5434189

[B25] France J. Jovanovic T. (2023). Human fear neurobiology reimagined: Can brain-derived biotypes predict fear-based disorders after trauma? *Neurosci. Biobehav. Rev.* 144:104988. 10.1016/J.NEUBIOREV.2022.104988 36470327 PMC10960960

[B26] Galatzer-Levy I. R. Bryant R. A. (2013). 636,120 ways to have posttraumatic stress disorder. *Perspect. Psychol. Sci.* 8 651–662. 10.1177/1745691613504115 26173229

[B27] Goodkind M. Eickhoff S. B. Oathes D. J. Jiang Y. Chang A. Jones-Hagata L. B. (2015). Identification of a common neurobiological substrate for mental illness. *JAMA Psychiatry* 72:305. 10.1001/JAMAPSYCHIATRY.2014.2206 25651064 PMC4791058

[B28] Gordon E. M. Laumann T. O. Adeyemo B. Huckins J. F. Kelley W. M. Petersen S. E. (2016). Generation and evaluation of a cortical area parcellation from resting-state correlations. *Cereb. Cortex* 26:288. 10.1093/CERCOR/BHU239 25316338 PMC4677978

[B29] Gratton C. Sun H. Petersen S. E. (2018). Control networks and hubs. *Psychophysiology* 55 10.1111/sy13032. 10.1111/PSYP.13032 29193146 PMC5811327

[B30] Harnett N. G. Finegold K. E. Lebois L. A. M. van Rooij S. J. H. Ely T. D. Murty V. P. (2022). Structural covariance of the ventral visual stream predicts posttraumatic intrusion and nightmare symptoms: A multivariate data fusion analysis. *Transl. Psychiatry* 12:321. 10.1038/S41398-022-02085-8 35941117 PMC9360028

[B31] Hoskins M. Pearce J. Bethell A. Dankova L. Barbui C. Tol W. A. (2015). Pharmacotherapy for post-traumatic stress disorder: Systematic review and meta-analysis. *Br. J. Psychiatry* 206 93–100. 10.1192/bjp.bp.114.148551 25644881

[B32] Johnsen G. E. Asbjørnsen A. E. (2008). Consistent impaired verbal memory in PTSD: A meta-analysis. *J. Affect. Disord.* 111 74–82. 10.1016/J.JAD.2008.02.007 18377999

[B33] Kessler R. C. Berglund P. Demler O. Jin R. Merikangas K. R. Walters E. E. (2005). Lifetime prevalence and age-of-onset distributions of DSM-IV disorders in the National Comorbidity Survey Replication. *Arch. Gen. Psychiatry* 62 593–602. 10.1001/ARCHPSYC.62.6.593 15939837

[B34] Kuhn E. Blanchard E. B. Hickling E. J. (2003). Posttraumatic stress disorder and psychosocial functioning within two samples of MVA survivors. *Behav. Res. Ther.* 41 1105–1112. 10.1016/S0005-7967(03)00071-8 12914811

[B35] Lewis C. Roberts N. P. Gibson S. Bisson J. I. (2020). Dropout from psychological therapies for post-traumatic stress disorder (PTSD) in adults: Systematic review and meta-analysis. *Eur. J. Psychotraumatol.* 11:1709709. 10.1080/20008198.2019.1709709 32284816 PMC7144189

[B36] Lindsay G. W. (2020). Attention in psychology, neuroscience, and machine learning. *Front. Comput. Neurosci.* 14:516985. 10.3389/FNCOM.2020.00029/BIBTEX PMC7177153 32372937

[B37] Luo T. Z. Maunsell J. H. R. (2019). Attention can be subdivided into neurobiological components corresponding to distinct behavioral effects. *Proc. Natl. Acad. Sci. U.S.A.* 116 26187–26194. 31871179 10.1073/pnas.1902286116 PMC6936701

[B38] McLaughlin K. A. Koenen K. C. Hill E. D. Petukhova M. Sampson N. A. Zaslavsky A. M. (2013). Trauma exposure and posttraumatic stress disorder in a national sample of adolescents. *J. Am. Acad. Child. Adolesc. Psychiatry* 52:815. 10.1016/J.JAAC.2013.05.011 23880492 PMC3724231

[B39] McTeague L. M. Huemer J. Carreon D. M. Jiang Y. Eickhoff S. B. Etkin A. (2017). Identification of common neural circuit disruptions in cognitive control across psychiatric disorders. *Am. J. Psychiatry* 174 676–685. 10.1176/APPI.AJP.2017.16040400 28320224 PMC5543416

[B40] Menon V. (2015). “Salience network,” in *Brain mapping: An encyclopedic reference*, Vol. 2 ed. Toga A. W. (Cambridge, MA: Academic Press), 597–611. 10.1016/B978-0-12-397025-1.00052-X

[B41] Mueller-Pfeiffer C. Schick M. Schulte-Vels T. O’Gorman R. Michels L. Martin-Soelch C. (2013). Atypical visual processing in posttraumatic stress disorder. *Neuroimage Clin.* 3 531–538. 10.1016/J.NICL.2013.08.009 24371791 PMC3871398

[B42] National Institute of Mental Health (2021). *Research domain criteria initiative: Attention.* Available online at: https://www.nimh.nih.gov/research/research-funded-by-nimh/rdoc/constructs/attention (accessed January 31, 2023).

[B43] Pergamin-Hight L. Naim R. Bakermans-Kranenburg M. J. van IJzendoorn M. H. Bar-Haim Y. (2015). Content specificity of attention bias to threat in anxiety disorders: A meta-analysis. *Clin. Psychol. Rev.* 35 10–18. 10.1016/J.CPR.2014.10.005 25462110

[B44] Petersen S. E. Posner M. I. (2012). The attention system of the human brain: 20 years after. *Annu. Rev. Neurosci.* 35 73–89. 10.1146/annurev-neuro-062111-150525 22524787 PMC3413263

[B45] Punski-Hoogervorst J. L. Engel-Yeger B. Avital A. (2023). Attention deficits as a key player in the symptomatology of posttraumatic stress disorder: A review. *J. Neurosci. Res*. 101 1068–1085. 10.1002/JNR.25177 36807926

[B46] Raichle M. E. (2015). The brain’s default mode network. *Annu. Rev. Neurosci.* 38 433–447. 10.1146/ANNUREV-NEURO-071013-014030 25938726

[B47] Ross M. C. Cisler J. M. (2020). Altered large-scale functional brain organization in posttraumatic stress disorder: A comprehensive review of univariate and network-level neurocircuitry models of PTSD. *Neuroimage Clin.* 27:102319. 10.1016/J.NICL.2020.102319 32622316 PMC7334481

[B48] Saito D. N. Fujisawa T. X. Yanaka H. T. Fujii T. Kochiyama T. Makita K. (2022). Development of attentional networks during childhood and adolescence: A functional MRI study. *Neuropsychopharmacol. Rep.* 42 191–198. 10.1002/NPR2.12246 35266330 PMC9216368

[B49] Scolari M. Seidl-Rathkopf K. N. Kastner S. (2015). Functions of the human frontoparietal attention network: Evidence from neuroimaging. *Curr. Opin. Behav. Sci.* 1 32–39. 10.1016/j.cobeha.2014.08.003 27398396 PMC4936532

[B50] Scott J. C. Matt G. E. Wrocklage K. M. Crnich C. Jordan J. Southwick S. M. (2015). A quantitative meta-analysis of neurocognitive functioning in posttraumatic stress disorder. *Psychol. Bull.* 141:105. 10.1037/A0038039 25365762 PMC4293317

[B51] Seeley W. W. (2019). The salience network: A neural system for perceiving and responding to homeostatic demands. *J. Neurosci.* 39 9878–9882. 10.1523/JNEUROSCI.1138-17.2019 31676604 PMC6978945

[B52] Seeley W. W. Menon V. Schatzberg A. F. Keller J. Glover G. H. Kenna H. (2007). Dissociable intrinsic connectivity networks for salience processing and executive control. *J. Neurosci.* 27:2349. 10.1523/JNEUROSCI.5587-06.2007 17329432 PMC2680293

[B53] Steenkamp M. M. Litz B. T. Hoge C. W. Marmar C. R. (2015). Psychotherapy for military-related PTSD: A review of randomized clinical trials. *JAMA* 314 489–500. 10.1001/JAMA.2015.8370 26241600

[B54] Susanty E. Sijbrandij M. van Dijk W. Srisayekti W. de Vries R. Huizink A. C. (2022). The effects of psychological interventions on neurocognitive functioning in posttraumatic stress disorder: A systematic review. *Eur. J. Psychotraumatol.* 13:2071527. 10.1080/20008198.2022.2071527 35957628 PMC9359170

[B55] Uddin L. Q. (2014). Salience processing and insular cortical function and dysfunction. *Nat. Rev. Neurosci.* 16 55–61. 10.1038/nrn3857 25406711

[B56] Uddin L. Q. Yeo B. T. T. Spreng R. N. (2019). Towards a universal taxonomy of macro-scale functional human brain networks. *Brain. Topogr.* 32:926. 10.1007/S10548-019-00744-6 31707621 PMC7325607

[B57] Vasterling J. J. Constans J. I. Brailey K. Sutker P. B. (1998). Attention and memory dysfunction in posttraumatic stress disorder. *Neuropsychology* 12 125–133. 10.1037/0894-4105.12.1.125 9460740

[B58] Vossel S. Geng J. J. Fink G. R. (2014). Dorsal and ventral attention systems: Distinct neural circuits but collaborative roles. *Neuroscientist* 20 150–159. 10.1177/1073858413494269 23835449 PMC4107817

[B59] Watkins L. E. Sprang K. R. Rothbaum B. O. (2018). Treating PTSD: A review of evidence-based psychotherapy interventions. *Front. Behav. Neurosci.* 12:258. 10.3389/fnbeh.2018.00258 PMC6224348 30450043

[B60] Yeo B. T. Krienen F. M. Sepulcre J. Sabuncu M. R. Lashkari D. Hollinshead M. (2011). The organization of the human cerebral cortex estimated by intrinsic functional connectivity. *J. Neurophysiol.* 106:1125. 10.1152/JN.00338.2011 21653723 PMC3174820

[B61] Zhou Y. Friston K. J. Zeidman P. Chen J. Li S. Razi A. (2018). The hierarchical organization of the default, dorsal attention and salience networks in adolescents and young adults. *Cereb. Cortex* 28 726–737. 10.1093/CERCOR/BHX307 29161362 PMC5929108

